# Rational Design of Superior Electrocatalysts for Water Oxidation: Crystalline or Amorphous Structure?

**DOI:** 10.1002/smsc.202100030

**Published:** 2021-08-03

**Authors:** Daqin Guan, Wei Zhou, Zongping Shao

**Affiliations:** ^1^ State Key Laboratory of Materials-Oriented Chemical Engineering College of Chemical Engineering Nanjing Tech University Nanjing 211800 China; ^2^ Department of Chemical Engineering WA School of Mines: Minerals, Energy and Chemical Engineering (WASM‐MECE) Curtin University Perth WA 6102 Australia

**Keywords:** amorphous structures, crystalline structures, crystalline–amorphous structures, water splitting

## Abstract

Crystalline, amorphous, and crystalline–amorphous materials have become three important electrode materials for the bottleneck oxygen‐evolving reaction (OER) in the promising hydrogen‐producing technology of water splitting. With the rapid development of in situ/ex situ characterizations, the understanding of active sites in electrocatalysts has been deepened via the structure–activity/stability relationships extracted from the observations on catalysts during/after the OER. Herein, the origins of changes in initial crystalline, amorphous, and crystalline–amorphous materials during/after the OER are systematically analyzed and the underlying variation effects on catalyst activity and stability are discussed based on recent representative studies, aiming at guiding OER catalyst design in the future.

## Graphical abstract

Crystalline, amorphous, and crystalline–amorphous materials have been explored for catalyzing the oxygen‐evolving reaction (OER). However, a systematic insight into the origins and effects of the behaviors on initial crystalline, amorphous, and crystalline–amorphous materials during/after the OER is still absent. Herein, it is attempted to offer a systematic viewpoint on these issues to guide the rational design of electrocatalysts.

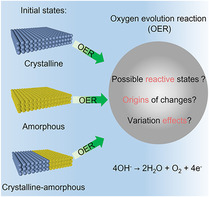

## 1 Brief History

**Figure 1:**
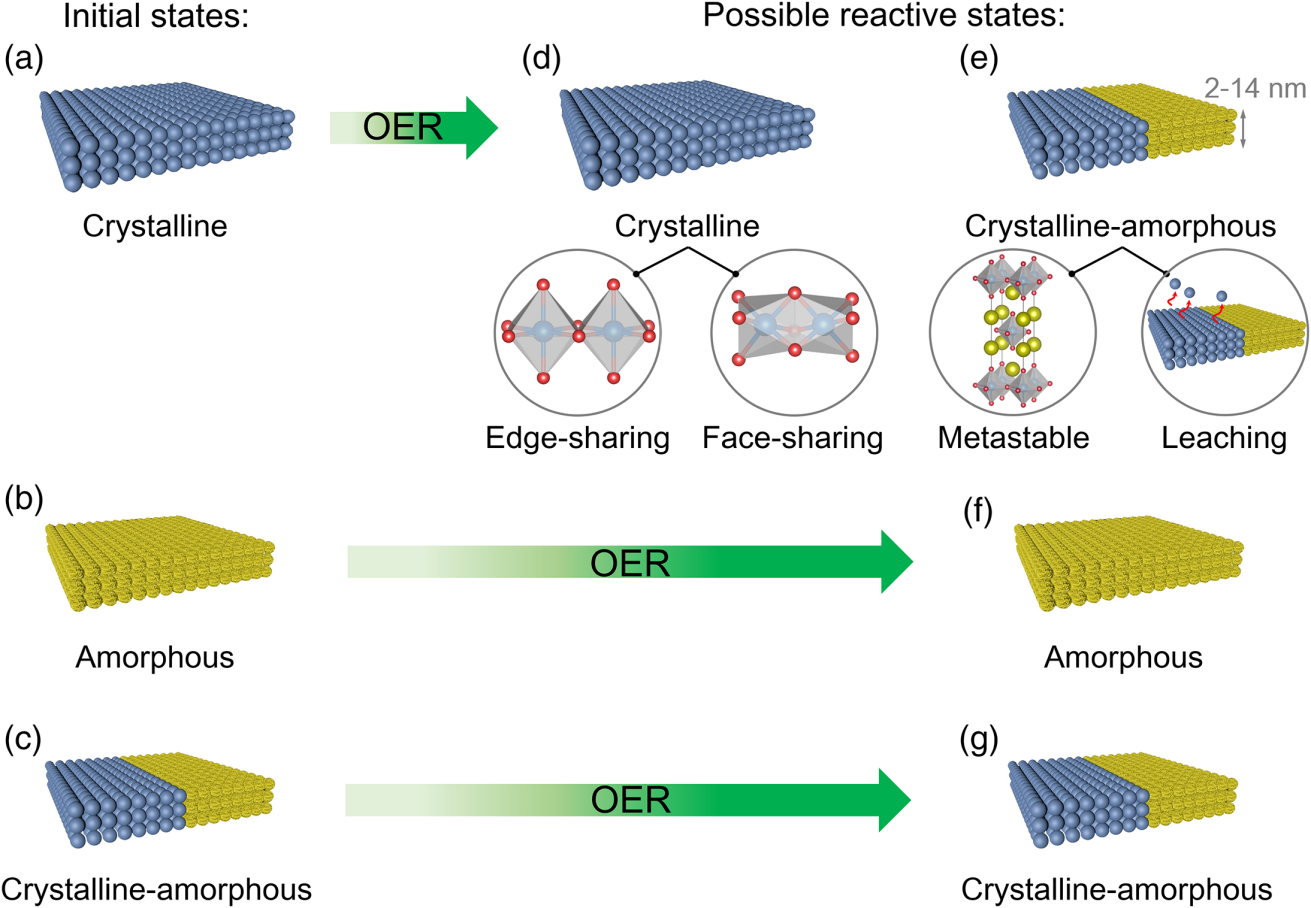
The effects of possible reactive states for initial crystalline, amorphous, and crystalline–amorphous structures. Initial a) crystalline, b) amorphous, and c) crystalline–amorphous structural states. Possible reactive d) crystalline and e) crystalline–amorphous states (reaction depth is 2–14 nm) for the initial crystalline structural state. Possible reactive f) amorphous and g) crystalline–amorphous states for initial amorphous and crystalline–amorphous structural states, respectively.

Utilizing electricity produced from wind, solar, hydro, and nuclear fusion to synthesize various C/N/O/H‐containing chemicals is one of the most promising sustainable pathways.^[^ ^1^ ^]^ Due to the merits of renewable electricity input, abundant water resources, and green synthesis processes, hydrogen production through water splitting is considered as one of the most fundamental and important parts in the electrochemical synthesis roadmap.^[^ ^2^, ^3^ ^]^ However, this technology suffers from high costs of electricity consumption (≈80% total cost) and the sluggish bottleneck of the oxygen‐evolving reaction (OER).^[^ ^3^, ^4^ ^]^ Therefore, developing highly efficient and durable OER electrocatalysts has become one of the biggest challenges in the application of water splitting. To date, three typical candidates, namely, crystalline, amorphous, and crystalline–amorphous materials (**Figure** **1**a–c and **Table** **1**), have been explored for catalyzing the OER. In addition, in situ/ex situ technologies have been widely applied in OER studies, pushing the establishment of structure–activity/stability trends based on the catalyst behaviors during/after the OER.

**Table 1:** The changes on initial crystalline, amorphous, and crystalline–amorphous materials during/after OER and their OER activity and stability

Materials	Liquid	Initial state	Reactive state	Overpotential	Stability
Edge‐sharing LiCo_0.8_Fe_0.2_O_2_^[^ ^39^ ^]^	0.1 m KOH	Crystalline	Crystalline	≈340 mV @ 10 mA cm^−2^_disk_ a)	10 h @ 10 mA cm^−2^_disk_
Edge‐sharing LiCoVO_4_^[^ ^40^ ^]^	1.0 m KOH	Crystalline	Crystalline	≈364 mV @ 5 mA cm^−2^_oxide_ b)	100 CV cycles
Edge‐sharing Ca_2_IrO_4_^[^ ^41^ ^]^	0.5 m H_2_SO_4_	Crystalline	Crystalline	≈350 mV @ 0.64 mA cm^−2^_oxide_	200 CV cycles
Face‐sharing 6H‐SrIrO_3_^[^ ^42^ ^]^	0.5 m H_2_SO_4_	Crystalline	Crystalline	248 mV @ 10 mA cm^−2^_disk_	30 h @ 10 mA cm^−2^_disk_
Face‐sharing Ba_3_TiIr_2_O_9_^[^ ^43^ ^]^	0.1 m HClO_4_	Crystalline	Crystalline	275 mV @ 10 mA cm^−2^_disk_	20 h @ 10 mA cm^−2^_disk_
Face‐sharing BaIrO_3_^[^ ^41^ ^]^	0.5 m H_2_SO_4_	Crystalline	Crystalline	≈350 mV @ 1.57 mA cm^−2^_oxide_	200 CV cycles
PrBaCo_2_O_6−*δ*_ (Co^3+/4+^)^[^ ^16^ ^]^	0.1 m KOH	Crystalline	Crystalline	≈330 mV @ 10 mA cm^−2^_oxide_	2 h @ 5 mA cm^−2^_disk_
PrBa_0.5_Sr_0.5_Co_1.5_Fe_0.5_O_6−*δ*_ (Co^3+/4+^)^[^ ^44^ ^]^	0.1 m KOH	Crystalline	Crystalline	358 mV @ 10 mA cm^−2^_disk_	12 h @ 10 mA cm^−2^_disk_
Ba_4_Sr_4_(Co_0.8_Fe_0.2_)_4_O_15_ (Co^3+/4+^)^[^ ^45^ ^]^	0.1 m KOH	Crystalline	Crystalline	340 mV @ 10 mA cm^−2^_disk_	10 h @ 10 mA cm^−2^_disk_
Na_0.67_CoO_2_ (Co^3+/4+^)^[^ ^46^ ^]^	0.1 m KOH	Crystalline	Crystalline	290 mV @ 10 mA cm^−2^_disk_	1000 CV cycles
Ba_0.35_Sr_0.65_Co_0.8_Fe_0.2_O_3−*δ*_ (Co^3+/4+^)^[^ ^4^ ^]^	0.1 m KOH	Crystalline	Crystalline	260 mV @ 10 mA cm^−2^_disk_	100 h @ 10 mA cm^−2^_disk_
ZnFe_0.4_Co_1.6_O_4_ (Co^3+/4+^)^[^ ^47^ ^]^	0.1 m KOH	Crystalline	Crystalline	≈384 mV @ 5 mA cm^−2^_disk_	5000 CV cycles
RP‐type La_2_Li_0.5_Ni_0.5_O_4_^[^ ^48^ ^]^	1.0 m KOH	Crystalline	Amorphous	≈396 mV @ 1 mA_disk_	30 CV cycles
Ba_0.5_Sr_0.5_Co_0.8_Fe_0.2_O_3−*δ*_ (Co^2+/3+^)^[^ ^24^ ^]^	0.1 m KOH	Crystalline	Amorphous	≈370 mV @ 3 mA cm^−2^_disk_	500 CV cycles
LaCo_0.8_Fe_0.2_O_3−*δ*_ (Co^0+/2+/3+^)^[^ ^50^ ^]^	0.1 m KOH	Crystalline	Amorphous	293 mV @ 10 mA cm^−2^_disk_	100 h @ 10 mA cm^−2^_disk_
NiFe Prussian blue analogues (Ni^2+^)^[^ ^51^ ^]^	1.0 m KOH	Crystalline	Amorphous	258 mV @ 10 mA cm^−2^_disk_	100 h @ 10 mA cm^−2^_disk_
La_1−*x*_Sr_*x*_CoO_3_ (Sr^2+^ leaching)^[^ ^53^ ^]^	0.1 m KOH	Crystalline	Amorphous	≈470 mV @ 1 mA cm^−2^_disk_	100 CV cycles
SnNiFe oxide (Sn^4+^ leaching)^[^ ^54^ ^]^	0.1 m KOH	Crystalline	Amorphous	350 mV @ 10 mA cm^−2^_disk_	≈5.6 h @ 10 mA cm^−2^_disk_
FeCoW hydroxide^[^ ^55^ ^]^	0.1 m KOH	Amorphous	Amorphous	191 mV @ 10 mA cm^−2^_disk_	550 h @ 30 mA cm^−2^_disk_
LaNiFe hydroxide^[^ ^56^ ^]^	1.0 m KOH	Amorphous	Amorphous	189 mV @ 10 mA cm^−2^_disk_	100 h @ 10 mA cm^−2^_disk_
RuTe_2_ alloy^[^ ^57^ ^]^	0.5 m H_2_SO_4_	Amorphous	Amorphous	245 mV @ 10 mA cm^−2^_disk_	24 h @ ≈7 mA cm^−2^_disk_
NiFe alloy^[^ ^58^ ^]^	1.0 m KOH	Amorphous	Amorphous	265 mV @ 10 mA cm^−2^_disk_	200 h @ 40 mA cm^−2^_disk_
CoFe oxide^[^ ^59^ ^]^	0.1 m KOH	Amorphous	Amorphous	≈402 mV @ 10 mA mg−^1^	N.A.c)
NiFeMo oxide^[^ ^60^ ^]^	0.1 m KOH	Amorphous	Amorphous	280 mV @ 10 mA cm^−2^_disk_	40 h @ 10 mA cm^−2^_disk_
F‐doped Co_2_B^[^ ^61^ ^]^	1.0 m KOH	Crystalline–amorphous	Crystalline–amorphous	320 mV @ 10 mA cm^−2^_disk_	35 h @ 100 mA cm^−2^_disk_
Ni_1.5_Sn^[^ ^62^ ^]^	1.0 m KOH	Crystalline–amorphous	Crystalline–amorphous	240 mV @ 10 mA cm^−2^_disk_	10 h @ 1.56 V vs RHEd)
CoV—Fe_0.28_^[^ ^63^ ^]^	1.0 m KOH	Crystalline–amorphous	Crystalline–amorphous	215 mV @ 10 mA cm^−2^_disk_	40 h @ 1.55 V vs RHE
Ru@FeCoNi LDH^[^ ^64^ ^]^	1.0 m KOH	Crystalline–amorphous	Crystalline–amorphous	205 mV @ 10 mA cm^−2^_disk_	48 h @ 10 mA cm^−2^_disk_

a) These OER current densities are normalized to the surface area of electrodes;

b) These OER current densities are corrected to the Brunauer–Emmett–Teller (BET) surface area;

c) N.A. = Not available;

d) RHE = Reversible hydrogen electrode.

The anodic OER of water splitting in alkaline solutions is 4OH^−^ → 2H_2_O + O_2_ + 4e^−^, which possesses the four‐electron transfer feature.^[^ ^1^, ^4^ ^]^ Compared with the two‐electron hydrogen‐evolving reaction (HER) processes in alkaline media (2H_2_O + 2e^−^ → H_2_ + 2OH^−^),^[^ ^5^, ^6^ ^]^ the four‐electron OER steps are much more difficult and thus the OER is regarded as a bottleneck reaction in water–alkali electrolyzers. The material candidates for alkaline OER are usually transition‐metal‐based compounds containing precious metals or non‐noble metals. Due to the high cost and scarce nature of precious‐metal resources, the widespread application of noble‐metal‐based materials may be hindered for future commercial industries. It is noteworthy that non‐precious‐metal‐based materials with advantages of low costs, abundant resources, rich physicochemical properties, and remarkable performance have been pushed to the forefront for catalyzing the alkaline OER over the last decades.^[^ ^7^, ^8^ ^]^ Specifically, non‐precious‐metal‐based materials show large structural and electronic structural degrees of freedom. For example, non‐precious‐metal‐based oxides possess extremely rich structures of simple oxides, perovskite oxides, perovskite‐type oxides, and spinel oxides, which allow abundant space groups and various element combinations in structural matrixes.^[^ ^7^, ^8^ ^]^ In addition, in terms of the electronic structures, non‐precious‐metal ions exhibit many unique spin, charge, coordination, and orbital properties. For instance, Co ions can show Co^2+^ with high‐spin (HS) state,^[^ ^9^ ^]^ Co^3+^ with HS,^[^ ^10^ ^]^ low‐spin (LS),^[^ ^11^ ^]^ and intermediate‐spin (IS)^[^ ^12^ ^]^ states, as well as Co^4+^ with LS^[^ ^13^ ^]^ and IS^[^ ^14^ ^]^ states. Benefiting from the rich structural and electronic information, non‐precious metal‐based compounds are widely explored in the alkaline OER.

As another critical achievement in alkaline OER studies during the past decades, activity/stability descriptor frameworks have been successfully established based on the relationships between the initial physicochemical properties of electrocatalysts and their OER performance to guide material design. For example, Suntivich et al.^[^ ^15^ ^]^ found that the intrinsic OER activity of 3* d* transition‐metal‐based perovskite oxides exhibits a volcano‐shaped trend with material *e*_*g*_ occupancy, where the Ba_0.5_Sr_0.5_Co_0.8_Fe_0.2_O_3−*δ*_ perovskite with an *e*_*g*_ occupancy of ≈1.2 climbs to the top of the volcano plot. Also, Grimaud et al.^[^ ^16^ ^]^ proposed that the O 2*p* band center neither too far nor too close from the Fermi level would contribute to the high OER activity and stability of double perovskites. Recently, Hong et al.^[^ ^17^ ^]^ reported that the charge‐transfer energy can serve as an efficient OER activity descriptor to rationalize different OER pathways on catalysts.

However, with the rapid development of in situ/operando techniques (such as optical microscopy, X‐ray diffraction, transmission electron microscopy, X‐ray photoelectron spectroscopy, and X‐ray absorption spectroscopy) and the increasing studies concerning the states of electrocatalysts after the alkaline OER,^[^ ^18^, ^19^, ^20^, ^21^ ^]^ previous structure–activity/stability relationships between the initial states of electrocatalysts and catalysis performance have been greatly challenged. For instance, subsequent studies showed that Ba_0.5_Sr_0.5_Co_0.8_Fe_0.2_O_3−*δ*_ perovskite oxide would undergo surface structural amorphization during/after the OER^[^ ^22^, ^23^ ^]^ and the Co^2+/3+^ ions in the Ba_0.5_Sr_0.5_Co_0.8_Fe_0.2_O_3−*δ*_ material would increase to higher Co valence states under the OER conditions by means of operando hard X‐ray absorption spectroscopy.^[^ ^24^ ^]^ Moreover, Zhou et al.^[^ ^25^ ^]^ observed that the Co valence and spin states in Li_2_Co_2_O_4_ spinel oxide (pure Co^3+^ in LS state) can transform into Co^3.4+^ in the LS state under OER voltages by using operando soft X‐ray absorption spectroscopy. Recently, Ham et al.^[^ ^26^ ^]^ reported that the octahedral Co^2+^ ions in CoSb_2_O_6_ trirutile would be oxidized to Co^3+^ ions and further to Co^4+^ ions under OER conditions. The structural/electronic structural variations of OER electrocatalysts during/after the OER will change the material *e*_*g*_ occupancy, orbital state, charge‐transfer energy value, and many other physicochemical properties. Therefore, establishing the structure–activity/stability relationships between the states of electrocatalysts during/after the OER and their performance may be a better and more accurate method to guide the material design.

Despite the aforementioned achievements, a systematic insight into the origins and effects of the behaviors of initial crystalline, amorphous, and crystalline–amorphous materials during/after the OER is still absent. We attempt to offer a systematic viewpoint on these issues to guide the rational design of three typical candidates.

## 2 Possible Change Origins and Structure–Performance Relationships

The molecular structure with long‐range ordering feature is defined as crystalline structure (Figure 1a). Many efficient strategies can be applied to moderate the physicochemical properties of crystalline structures, such as phase engineering (including some special phases),^[^ ^27^, ^28^, ^29^ ^]^ defect chemistry,^[^ ^30^, ^31^ ^]^ corrosion engineering,^[^ ^32^, ^33^ ^]^ strain engineering,^[^ ^34^, ^35^ ^]^ and many other methodologies.^[^ ^8^, ^36^ ^]^ Generally, initial crystalline materials could maintain crystallinity or become partially amorphous during/after the OER (Figure 1d,e and Table 1), which is determined by the initial physicochemical properties of the crystalline materials. It is worth mentioning that the OER depth was reported to be 2–14 nm (Figure 1).^[^ ^4^, ^37^, ^38^ ^]^ For initial crystalline materials that can keep crystalline structures during/after the OER (Figure 1d and Table 1), these compounds usually possess stable edge‐sharing/face‐sharing structure units or high‐valence active metal ions. For the former, benefitting from the two (for edge‐sharing motifs) or three (for face‐sharing motifs) stable shared bonds, these edge‐sharing/face‐sharing crystalline materials can be stable during/after the OER (Figure 1d), contributing to their good OER durability, such as edge‐sharing LiCo_0.8_Fe_0.2_O_2_,^[^ ^39^ ^]^ edge‐sharing LiCoVO_4_,^[^ ^40^ ^]^ edge‐sharing Ca_2_IrO_4_ (stable even in acidic OER),^[^ ^41^ ^]^ face‐sharing 6H‐SrIrO_3_ (stable even in acidic OER),^[^ ^42^ ^]^ face‐sharing Ba_3_TiIr_2_O_9_ (stable even in acidic OER),^[^ ^43^ ^]^ and face‐sharing BaIrO_3_ (stable even in acidic OER).^[^ ^41^ ^]^ For the latter, initial crystalline materials with high‐valence active metal ions can relieve the effects of OER oxidation currents to maintain crystalline states and further enhance OER stabilities, as reported in the cases of PrBaCo_2_O_6−*δ*_ (Co^3+/4+^),^[^ ^16^ ^]^ PrBa_0.5_Sr_0.5_Co_1.5_Fe_0.5_O_6−*δ*_ (Co^3+/4+^),^[^ ^44^ ^]^ Ba_4_Sr_4_(Co_0.8_Fe_0.2_)_4_O_15_ (Co^3+/4+^),^[^ ^45^ ^]^ Na_0.67_CoO_2_ (Co^3+/4+^),^[^ ^46^ ^]^ hybrid Ba_0.35_Sr_0.65_Co_0.8_Fe_0.2_O_3−*δ*_ (Co^3+/4+^),^[^ ^4^ ^]^ and ZnFe_0.4_Co_1.6_O_4_ (Co^3+/4+^)^[^ ^47^ ^]^ materials.

In terms of initial crystalline compounds that are greatly affected by OER oxidation currents, these material structures would be destroyed and turn partially amorphous during/after the OER (Figure 1e and Table 1). Over the past years, these materials have been widely reported, where the reconstructed amorphous surface is recognized as the real active sites. Thus, these materials are also called “precatalysts.” The origins of this transformation during/after the OER on these precatalysts can be ascribed to three major factors: 1) The initial crystalline structure is unstable; 2) the valence of the active metal ion is low; and 3) ion leaching behaviors exist on these materials.

For factor (1), some crystalline structures are unstable, namely, in the metastable state. Under the functions of foreign OER potentials, the surface of these crystalline compound structures would collapse and recombine into amorphous structures. For example, Ruddlesden–Popper (RP) oxides with unstable rock‐salt layers show metastable phases and tend to become amorphous under electrochemical potentials (Figure 1e), for example, La_2_Li_0.5_Ni_0.5_O_4_^[^ ^48^ ^]^ and La_0.5_Sr_1.5_MnO_4_^[^ ^49^ ^]^ oxides. Interestingly, Yang et al.^[^ ^48^ ^]^ found that the formed amorphous layers on La_2_Li_0.5_Ni_0.5_O_4_ crystalline oxide after the same OER cycles in KOH solutions with different pH values are quite different, where the surface degradation depends on the pH and increased thickness of amorphous layers is formed with increasing pH values. For factor (2), if the initial valence of the active metal ions in crystalline materials is low, then these ions tend to be oxidized to higher valence states under OER oxidation currents, leading to the crystal oscillations and the formation of amorphous surfaces, such as Ba_0.5_Sr_0.5_Co_0.8_Fe_0.2_O_3−*δ*_ (Co^2+/3+^),^[^ ^24^ ^]^ H_2_/Ar‐reduced LaCo_0.8_Fe_0.2_O_3−*δ*_ (Co^0+/2+/3+^),^[^ ^50^ ^]^ and NiFe Prussian blue analogues (Ni^2+^).^[^ ^51^ ^]^ In terms of factor (3), some elements in precatalysts would leach into liquid under OER potentials, resulting in the loss of surface crystalline structure and the growth of an amorphous surface (Figure 1e). For example, S/P/N elements in S/P/N‐containing materials tend to leach into the electrolyte and these material surfaces would transform into amorphous oxides/hydroxides.^[^ ^52^ ^]^ In addition, the weak ionic bonds in some oxides would break under the functions of OER currents, leading to material ion leaching behaviors and the generation of amorphous surfaces, as shown in the cases of Ba_0.5_Sr_0.5_Co_0.8_Fe_0.2_O_3−*δ*_ (Ba^2+^, Sr^2+^ leaching),^[^ ^22^ ^]^ La_1−*x*_Sr_*x*_CoO_3_ (Sr^2+^ leaching),^[^ ^53^ ^]^ and SnNiFe oxide (Sn^4+^ leaching).^[^ ^54^ ^]^ Rish et al.^[^ ^22^ ^]^ found that the amorphous edge‐sharing surface motifs would generate on the Ba_0.5_Sr_0.5_Co_0.8_Fe_0.2_O_3−*δ*_ crystalline oxide after Ba^2+^ and Sr^2+^ ion leaching due to the functions of OER currents, where these formed amorphous surface structures may be the real active sites on this material for the OER.

The aforementioned reconstruction behaviors in precatalysts during/after the OER could bring about beneficial changes of better hydrophilia and more exposed active sites to accelerate the OER kinetics and further promote OER activities. Benefiting from these advantages of amorphous structures, amorphous materials have been brought to the forefront for catalyzing the OER in recent years.

The molecular structure with only short‐range ordering is called amorphous structure (Figure 1b). These structures usually possess disordered atomic arrangement, rich dangling bonds and high surface areas to expose more active sites and further boost the OER activities. Moreover, initial amorphous materials can keep amorphous states during/after the OER (Figure 1f and Table 1), which is different from the variation of precatalysts discussed earlier. Thus, amorphous materials have been hotly studied in recent years, such as amorphous FeCoW hydroxide,^[^ ^55^ ^]^ amorphous LaNiFe hydroxide,^[^ ^56^ ^]^ amorphous RuTe_2_ alloy,^[^ ^57^ ^]^ amorphous NiFe alloy,^[^ ^58^ ^]^ amorphous CoFe oxide,^[^ ^59^ ^]^ and amorphous NiFeMo oxide.^[^ ^60^ ^]^

Earlier, we discussed that the amorphous structure can improve the OER activity while the stable crystalline structure is able to steadily catalyze the OER. Therefore, to simultaneously realize good activity and stability on OER electrocatalysts, initial crystalline–amorphous materials have been developed in very recent studies (Figure 1c and Table 1). Similar to amorphous structures, crystalline–amorphous materials can usually maintain initial crystalline–amorphous states during/after the OER (Figure 1g and Table 1). As expected, such new materials can not only show good OER activity but also exhibit fine OER durability to fulfill the requirements of a catalyst, which can be evidenced by the studies of crystalline–amorphous F‐doped Co_2_B,^[^ ^61^ ^]^ crystalline–amorphous Ni_1.5_Sn at the trimetallic phosphate matrix,^[^ ^62^ ^]^ crystalline–amorphous CoV—Fe_0.28_,^[^ ^63^ ^]^ and a crystalline–amorphous FeCoNi layered double‐hydroxide‐supported single‐Ru‐atom catalyst.^[^ ^64^ ^]^ In these cases, the crystalline–amorphous phase boundaries were reported to be the active sites in the crystalline–amorphous materials for the OER.

It is worth noting that some materials can be quite stable for the OER, whereas some can only steadily catalyze the OER for a short time (Table 1). This triggers an open question that what are the key factors to affect material OER stability. Here, through combining the detailed analysis in the aforementioned cases, we can conclude that two main conditions may endow materials with good OER stability: 1) The initial states of materials are stable themselves as shown in the case of some crystalline materials with edge‐sharing/face‐sharing structural motifs^[^ ^39^, ^42^, ^43^ ^]^ or high‐valence active metal ions^[^ ^4^, ^44^, ^45^, ^46^, ^47^ ^]^ and some amorphous^[^ ^55^, ^56^, ^58^ ^]^ or partially amorphous^[^ ^61^, ^62^, ^63^, ^64^ ^]^ material structures with short‐range orderings, which can relieve the function of OER oxidation currents to keep the initial states and show good OER stabilities. 2) The initial states of electrocatalysts can undergo a fast surface structural reconstruction along with the rapid formation of a stable reactive state, which can be supported by some cases of initial crystalline materials with stable reconstructed reactive surface structures for the OER.^[^ ^50^, ^51^ ^]^

## 3 Challenges and Opportunities

To sum up, according to recent representative studies, we have systematically discussed the origins for the different behaviors of initial crystalline, amorphous, and crystalline–amorphous materials during/after the OER. Also, we have established the possible relationships between the physicochemical properties of three candidates during/after the OER and their OER performance. However, some issues still remain for the three typical catalysts, which call for further investigations, for example: 1) For stable crystalline structures with good OER stability, one open question is how to design the optimum initial states or introduce effective strategies to break through their activity bottleneck; 2) whether the surface reconstruction/amorphization behaviors on OER precatalysts would terminate and what are the underlying termination mechanisms; 3) as the amorphous phases during/after the OER are usually low‐conductivity hydroxides with a soluble feature, therefore, how to design conductive and solution‐resistant amorphous structures with remarkable OER stability; 4) the development of the relatively new crystalline–amorphous material system is still lagging compared with that of the other two types of materials, and thus, more substantial efforts should be devoted to finding controllable and facile synthesis methods and understanding materials’ behavior during/after the OER to unravel their OER catalysis mechanisms; 5) also, developing in situ/operando advanced light sources and electron‐based strategies as well as alternative available techniques (e.g., optical microscopy) is key to having deep insights into the behaviors and mechanisms of these three typical catalyst surfaces during the reaction.

We hope this article can offer some useful insights into the rational design of crystalline, amorphous, and crystalline–amorphous materials in the future.

## Conflict of Interest

The authors declare no conflict of interest.
